# Biological Properties of Heparins Modified with an Arylazopyrazole-Based Photoswitch

**DOI:** 10.1021/acs.jmedchem.2c01616

**Published:** 2023-01-19

**Authors:** Marta Stolarek, Aleksandra Pycior, Piotr Bonarek, Małgorzata Opydo, Elzbieta Kolaczkowska, Kamil Kamiński, Andrzej Mogielnicki, Krzysztof Szczubiałka

**Affiliations:** † Faculty of Chemistry, Jagiellonian University, Gronostajowa 2, 30-387 Krakow, Poland; ‡ Faculty of Biochemistry, Biophysics and Biotechnology, Jagiellonian University, Gronostajowa 7, 30-387 Krakow, Poland; § Laboratory of Experimental Hematology, Institute of Zoology and Biomedical Research, Faculty of Biology, Jagiellonian University, Gronostajowa 9, 30-387 Krakow, Poland; ∥ Department of Pharmacodynamics, Medical University of Bialystok, Mickiewicza 2c, 15-089 Bialystok, Poland

## Abstract

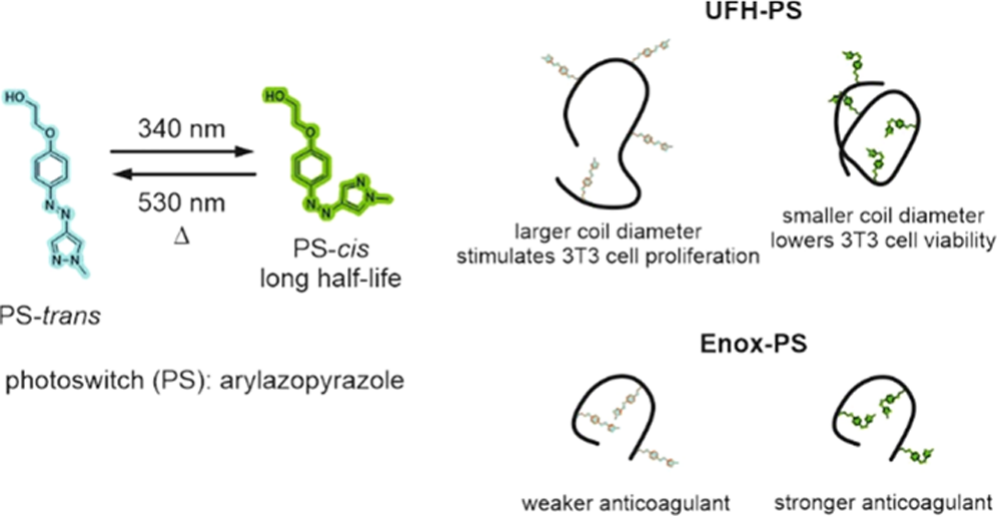

Unfractionated heparin (UFH) and enoxaparin (Enox) were substituted with a photoswitch (PS) showing quantitative *trans–cis* and *cis–trans* photoisomerizations. Long half-life of the *cis* photoisomer enabled comparison of the properties of heparins substituted with both PS photoisomers. Hydrodynamic diameter, *D*_h_, of UFH-PS decreased upon *trans–cis* photoisomerization, the change being more pronounced for UFH-PS with a higher degree of substitution (DS), while *D*_h_ of Enox-PS did not significantly change. The anticoagulative properties of substituted heparins were significantly attenuated compared to non-substituted compounds. The interaction of UFH-PS with HSA, lysozyme, and protamine was studied with ITC. Under serum-free conditions, UFH-PS-*trans* with a high DS stimulated proliferation of murine fibroblasts, while UFH-PS-*cis* decreased the viability of these cells. Under serum conditions, both UFH-PS-*cis* and UFH-PS-*trans* decreased cell viability, the reduction for UFH-PS-*cis* being higher than that for UFH-PS-*trans*. Neither Enox-PS-*trans* nor Enox-PS-*cis* influenced the viability at concentrations prolonging aPTT, while at higher concentrations their cytotoxicity did not differ.

## Introduction

Heparin is one of the oldest drugs in continuous use, it has been commercially available since the 1920s^1^ and it is widely used in clinics as an anticoagulant since 1937.^2^ In clinical practice it is applied in two main forms, i.e., as an unfractionated heparin (UFH) and as many variants of low-molecular-weight heparin (LMWH). The latter are obtained by the depolymerization of UFH with various methods therefore they differ in anticoagulant profiles, pharmacokinetic properties, and dosage regimens.^3^ LMWHs are generally considered to be safer than UFH because of their lower risk of hemorrhage and other adverse effects, do not require monitoring of anticoagulant activity, are cleared through kidneys, can be administered subcutaneously once daily, and show more predictable pharmacodynamics. On the other hand, UFH may be infused intravenously so it can be administered only under hospital conditions.

Except for being a mainstay anticoagulant, heparin shows many other activities, which have revived interest in its biomedical applications.^4−10^ It shows anti-inflammatory action, which may be used in the treatment of arthritis,^11^ bronchial asthma,^12^ pancreatitis,^13^ ulcerative colitis,^14^ and sepsis.^15^ Antiangiogenetic activity of heparin, which results from its ability to bind FGF and VEGF growth factors, may be applied to inhibit tumor angiogenesis in the treatment of cancer,^16^ while its anticoagulant activity is beneficial in fighting cancer-associated thrombosis.^17^ Heparin and insulin activate lipoprotein lipase leading to a decreased level of plasma triglycerides. This antihyperlipidemic activity can be used in the safe treatment of acute pancreatitis induced by hypertriglyceridemia.^18^ Moreover, heparin exerts antimicrobial activity with a wide spectrum of antiviral, antibacterial, and antiprotozoal actions. It is able to inhibit many viruses, including HSV-1,^19^ HPV,^20^ IV,^21^ HIV,^22^ ZIKV,^23^ and DENV,^24^ by directly interacting with the virus proteins or cellular receptors. Importantly, the recent intensive research directed to fight the COVID-19 pandemic has shown that its anticoagulant action combined with anti-inflammatory activity could decrease mortality in COVID-19 patients with sepsis-induced hypercoagulation.^25^ Enoxaparin (Enox), one of the most frequently used LMWHs, was found to bind to the SARS-CoV-2 spike glycoprotein and to strongly inhibit infection.^26^ By reducing the iron level in macrophages it also inhibits *Mycobacterium tuberculosis,*(27) while its selective binding to erythrocytes infected by *Plasmodium* may be used in the treatment of malaria.^28^ Finally, the effects of heparin such as a decrease in amyloid peptide production and the acceleration of its clearance, inhibition of tau phosphorylation, and reduction of inflammation may help in developing novel drugs in the treatment of Alzheimer’s disease.^29,30^

This list of beneficial actions of heparin is by no means complete. Their multitude makes it a potentially very versatile drug, however, its effects may mutually exclude its application. In particular, its strong anticoagulant activity may hinder its use in indications unrelated to pathological coagulation due to the risk of hemorrhage. Therefore, finding a way to attenuate or strengthen the selected physiological effect of heparin may open new perspectives for its clinical applications.

One of the possibilities to achieve selective, efficient, and safe control over the action of drugs and biomolecules is offered by the photopharmacological approach.^31−34^ Its principle is based on the application of photoactive compounds able to undergo irreversible photodissociation (photocages) or reversible photoisomerization (photoswitches, PSs). The PSs can be attached to their targets, such as drugs, proteins, ion channels, enzymes, etc., with covalent or noncovalent bonds.^32^ The photoisomerization of the PS results in a change in its geometry and size, which is expected to induce a significant change in the biological activity of the system into which it is incorporated, e.g., a drug.

To be of practical use in photopharmacology, a PS must conform to several stringent requirements. Both its photoisomers should significantly differ in their geometry, size, and physicochemical properties (e.g., dipole moment). The wavelengths used to produce them should be strongly absorbed, they should preferably lie in the phototherapeutic window (600–1000 nm)^35^ to minimize scattering and absorption by endogenous biomolecules and water, and the photoisomerization yield should be quantitative, or at least significant. Both photoisomers should be thermally stable for a time relevant to the drug pharmacokinetics. Finally, the photoswitch should be water-soluble and biologically stable, and its metabolites should not be toxic. Fulfilling all of these requirements simultaneously is difficult and an ideal photoswitch does not exist yet. However, there is a constant progress in the development of novel photoswitchable molecules, which more and more closely approach such a perfect PS. Of particular interest as PSs are the derivatives of arylazopyrazoles (AAPs),^36,37^ which may be considered as azobenzenes, in which one phenyl ring is replaced with a pyrazolyl ring. Therefore, a novel AAP-based compound was selected as a PS in this study.

This paper describes the study on heparin (both UFH and LMWH) derivatives obtained by the functionalization of heparin carboxyl groups with a PS moiety. The aim was to find out if physicochemical properties of heparin modified in this way, and consequently its biological activity, such as anticoagulative properties, interaction with proteins and cytotoxicity, change, and which of them, if any, can be controlled with light. To the best of our knowledge, research on the photocontrol of the biological activity of heparin has not been carried out so far. The positive answer obtained in this research suggests that with this approach it may be possible to gain photocontrol over other important pharmacological properties of heparin and possibly other biomacromolecules.

## Results and Discussion

### Synthesis and Photochemical Properties of the Photoswitch

**Figure 1:**
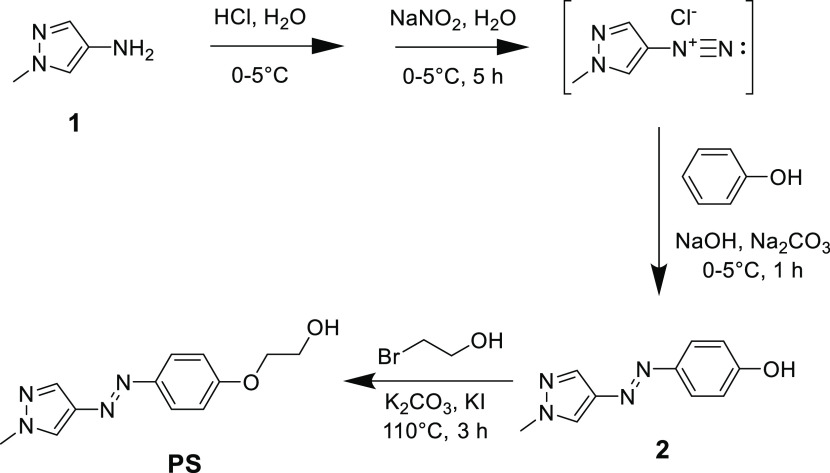
Synthesis of the photoswitch (PS).

The photoswitch (PS) used in the present study to functionalize heparins was an ether derivative of arylazopyrazole (AAP). Pyrazolylazophenyl ethers undergo *trans–cis* and reverse photoisomerizations when irradiated with near UV and visible light, respectively. They are known to show several advantages over other photoswitchable compounds, azobenzenes in particular.^38^ Importantly, *cis* photoisomers of pyrazolylazophenyl ethers show remarkable thermal stability (up to 3 months in organic solvents at RT). Moreover, they show almost quantitative conversion between both isomers, i.e., when irradiated with 365 and 530 nm light they reach a photostationary state (PSS) composed almost exclusively of *cis* and *trans* isomers, respectively. These two features, i.e., thermal stability of the *cis* isomer and quantitative photoconversion in both directions, are very difficult to achieve simultaneously. The PS was synthesized in two steps (Figure 1) based on the modified literature procedure.^38^

**Figure 2:**
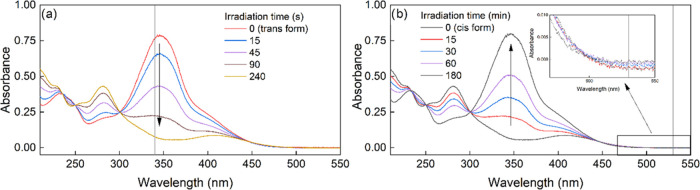
UV–vis spectra of PS-*trans* irradiated in water with 340 nm light, (a) indicating a quantitative conversion to PS-*cis*, which was then irradiated with 530 nm light and quantitatively converted back to PS-*trans* (b). Vertical lines indicate the irradiation wavelengths and the arrows indicate the direction of spectral changes. Note that the irradiation times are in seconds and minutes for *trans–cis* and *cis–trans* photoisomerizations, respectively. As shown in the inset in panel (b), absorption intensity of PS-*cis* at 530 nm is very low but still higher than that of PS-*trans* explaining the occurrence of *cis–trans* photoisomerization and its rate is slower than that of *trans–cis* photoisomerization.

In the first step, phenolic AAP derivative **1** was obtained and in the second step, its hydroxyl group was functionalized with the hydroxyethyl group, which was meant to be used to attach PS to heparin *via* the esterification reaction. On one hand, the chain of this group was short enough to make the molecule soluble in water and long enough to separate the terminal hydroxyl group from the phenol ring and avoid its potentially unfavorable influence on the advantageous photochemical properties of PS. Indeed, the PS obtained was soluble enough in water to yield measurable UV–vis spectra (Figure 2). They confirm that quantitative *trans–cis* and *cis–trans* photoconversions could be achieved, although due to the differences in the absorption intensity at the irradiation wavelengths, they occurred at a very different rate. To induce *trans–cis* photoisomerization 400 nm (thus visible) light could be also used, although it was not quantitative and took a longer time.

It was important to find out if the long lifetime of the *cis* isomer of PS was retained in aqueous media at the physiological temperature of 37 °C. Based on the rate of *cis–trans* conversion of PS measured using UV–vis spectra (data not shown) the half-life of PS-*cis* at room temperature, τ_1/2_, was 990 h (about 41 days), which at 37 °C was significantly shortened to about 224 h (about 9 days). This half-life is, however, long enough for this PS to be usable in photopharmacology.

### Synthesis of Photoswitchable Heparins

**Figure 3:**
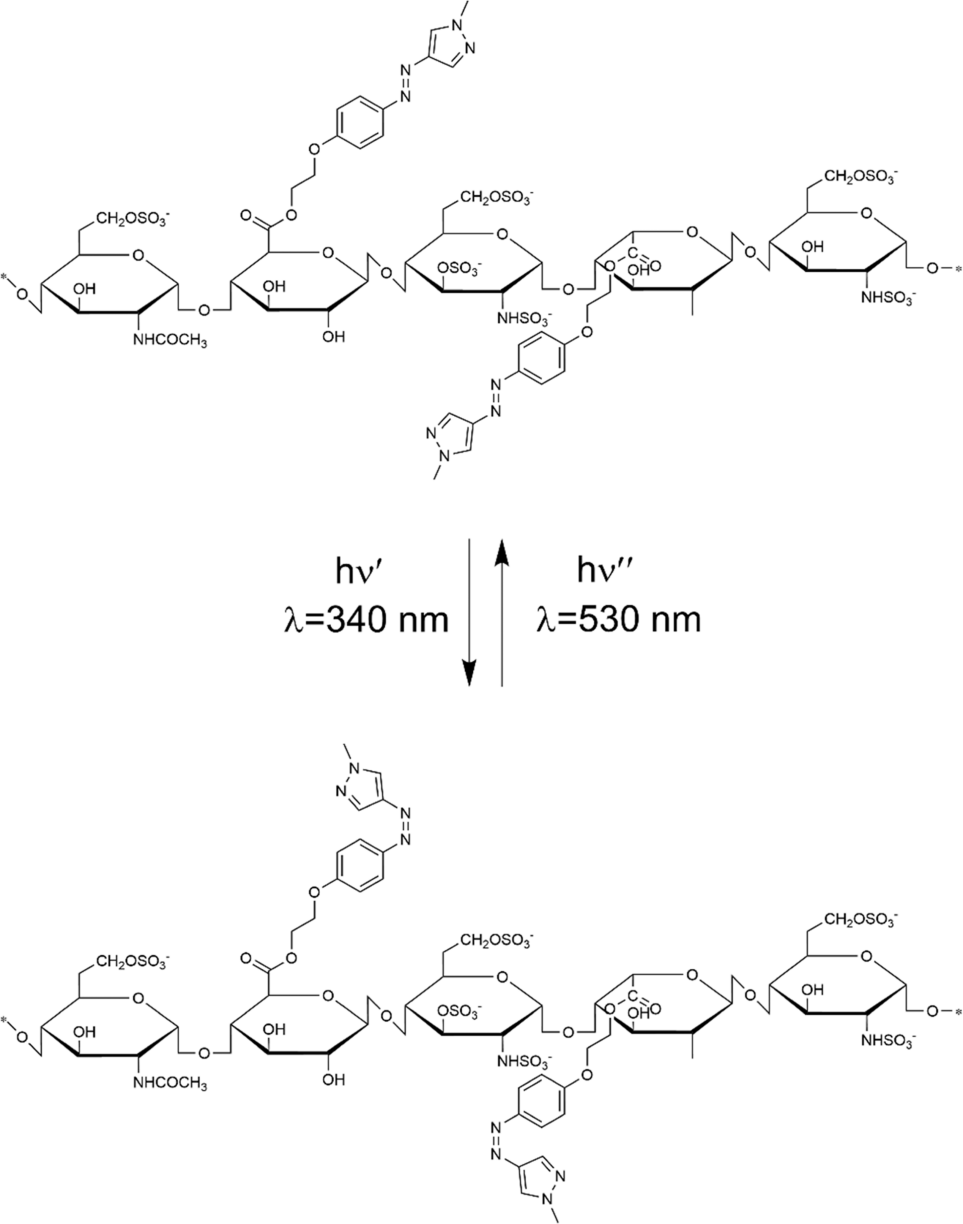
Structure and photoswitching of UFH/Enox substituted with PS.

Functionalization of heparins with PS was achieved by the esterification reaction between the uronic acid carboxyl groups of heparins and the hydroxyl groups of PS (Figure 3).

Heparins are not soluble in organic solvents, while PS is only moderately soluble in water. Therefore, to be able to carry out an efficient esterification reaction in an organic solvent the studied heparins were first converted into respective ammonium salts soluble in organics using Hyamine 1622^39^ (Figure S1). To obtain UFH with different degrees of substitution (DS) with PS, the esterification reaction was carried out under different conditions, including various **4**/PS mass ratios, DCC mass, and reaction time. Seven ester derivatives of UFH and one of Enox were obtained (Table 1).

**Table 1:** Reaction Conditions

	UFH-PS							
parameter	UFH-PS1	UFH-PS2	UFH-PS3	UFH-PS4	UFH-PS5	UFH-PS6	UFH-PS7	Enox-PS
PS (mg)	20	50	70	520	140	140	140	2000
**4** (mg)	250	250	250	500	430	430	430	1020
PS/**4** mass ratio	0.08	0.20	0.28	1.04	0.32	0.32	0.32	1.96
DCC (mg)	594	594	594	1188	1188	1188	1188	2380
PS/DCC mass ratio	0.034	0.084	0.12	0.44	0.12	0.12	0.12	0.84
reaction time (h)	48	48	48	121	48	96	168	96
yield (mg)	44	39	40	65	34	66	58	180
DS (μg/mg)	1.05	1.35	1.77	11.6	3.6	4.0	4.0	3.9

**Figure 4:**
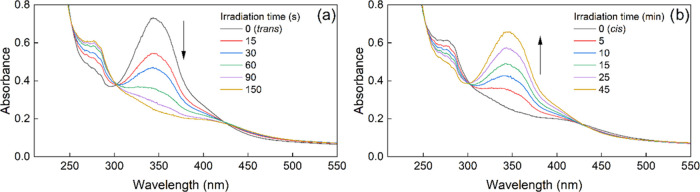
UV–vis absorption spectra of UFH-PS6 in water irradiated at (a) 340 nm indicating photoisomerization of UFH-PS3-*trans* into UFH-PS3-*cis*, which was then irradiated with (b) 530 nm light indicating reverse photoisomerization (*c* = 3.0 mg/mL, RT).

In the UV–vis spectra of all products, an absorption band with a maximum at 342 nm was found (Figure 4) proving successful substitution. Substitution could be also confirmed by the comparison of the IR spectra of Enox, Enox-PS, and PS (Figure S2). The UV–vis spectra were used to evaluate the degree of substitution (DS) of relevant heparin with PS (Table 1). Since the products UFH-PS5, UFH-PS6, and UFH-PS7 had similar DS, only UFH-PS6 was used in further studies. UFH-PS was insoluble in ethanol and soluble in water and phosphate-buffered saline (PBS) at a concentration of at least 3 mg/mL. Enox-PS turned out to be insoluble in water, however, it was soluble in PBS and in 5% v/v DMF in water at a concentration of at least 1 mg/mL.

### Photoswitching of Heparins

Irradiation of the aqueous solutions of UFH-PS and Enox-PS with 340 nm light resulted in the occurrence of *trans–cis* isomerization of the PS attached (Figure 3), which was accompanied by a decrease in the intensity of the 342 nm absorption band of PS substituents within up to a few minutes (Figure 4a). As expected, the *cis–trans* photoisomerization under 530 nm light took a much longer time (60 min), due to low absorption at this wavelength (Figure 4b).

Qualitatively the same results were obtained for photoswitching of Enox-PS (data not shown). It was then verified if the attachment of PS to the heparin chain influenced the lifetime of its *cis* photoisomer at different temperatures (Table 2).

**Table 2:** Values of the First Order Rate Constant of the Thermal *cis–trans* Isomerization of PS Free and Attached to Enox and Corresponding Half-Lives

	rate constant, *k* (1/h)		τ_1/2_ (h)	
temperature (C°)	PS	Enox-PS	PS	Enox-PS
RT	0.0007	0.0029	990	239
37	0.0031	0.0032	224	216
45		0.0066		105

The half-life of PS-*cis* in Enox-PS at 37 °C is about 9 days, much longer than the elimination half-life of UFH (30, 60, and 150 min for 25,^41^ 100,^42^ and 400 U/kg^43^ doses, respectively). In contrast to UFH, the elimination half-life of Enox is much longer (5–7 h) and is independent of the dose,^44^ but still significantly shorter than the half-life of Enox-PS-*cis*. Assuming that the half-life of PS-*cis* in Enox-PS-*cis* and UFH-PS-*cis* does not differ significantly this indicates that both UFH and Enox functionalized with PS-*cis* would be completely eliminated before their PS substituent would turn back into the *trans* configuration. This may be of practical significance as, on one hand, it allows the administration of either *trans* or *cis* forms of UFH-PS and Enox-PS. It also allows to show whether these forms exhibit different biological activities (e.g., anticoagulative action or cytotoxicity, see below) and, if so, to photoswitch these heparins in solution or *in vitro*/*in vivo* to change their activity.

### Influence of Photoswitching on the Conformation of the Heparin Chain in Solution

**Figure 5:**
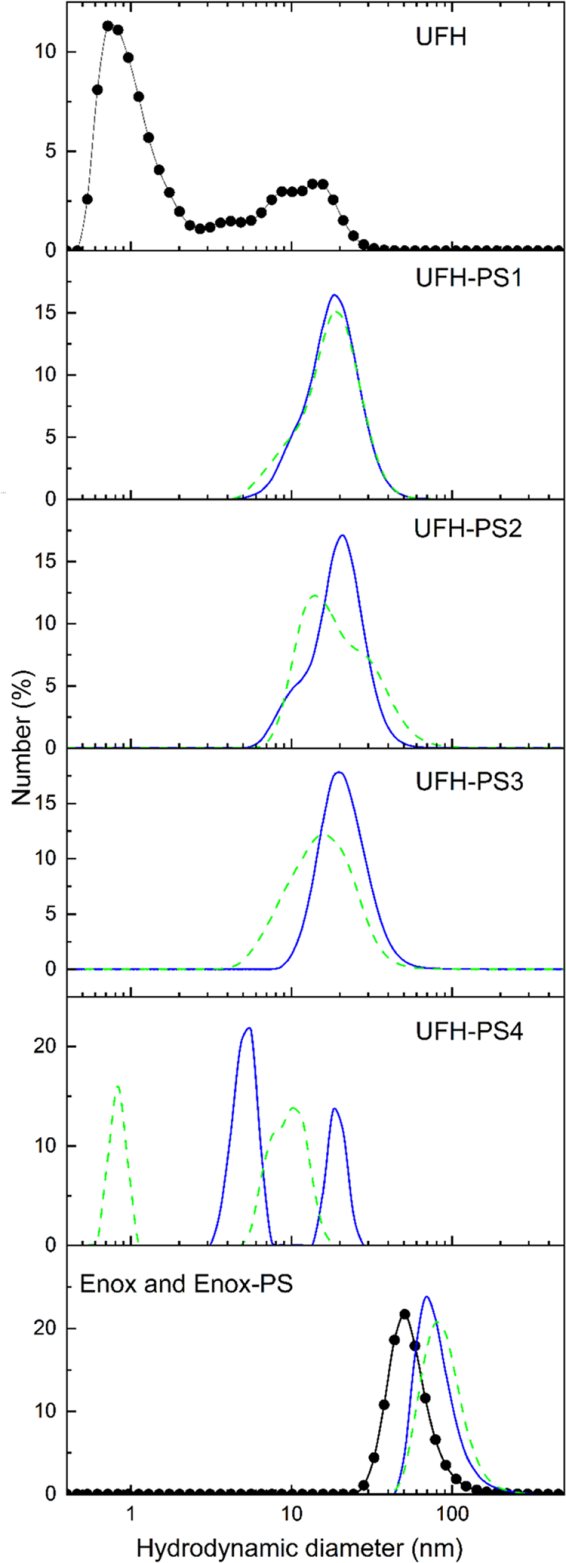
Hydrodynamic diameter, *D*_h_, distributions for UFH and UFH-PS with different DS. The respective distributions for Enox and Enox-PS are shown in the last panel (*c* = 0.4 mg/mL in PBS). The plots for heparins with PS in *trans* and *cis* configuration are shown as solid and dashed lines, respectively, while distributions for unsubstituted UFH and Enox are shown in the first and last panels, respectively (full circles).

Dynamic light scattering (DLS) measurements were performed to find out if the hydrodynamic diameter, *D*_h_, of the chains of heparin substituted with PS changes upon photoisomerization. Such changes would be an indication that their physiological activity may change as a result of PS photoisomerization. The number-average distributions for UFH/UFH-PS and Enox/Enox-PS are shown in Figure 5. DLS measurements showed that UFH is, as expected, a very inhomogeneous polymer with bimodal distribution of chain *D*_h_ with both modes at 0.7 and 13 nm, respectively. UFH substituted with the lowest amount of PS (UFH-PS1) had a much narrower distribution of chain *D*_h_ in PBS with a maximum at about 18 nm. The DS of UFH-PS1 turned out to be too low to result in the significant difference between the distributions of UFH-PS1-*trans* and UFH-PS1-*cis* chain diameters. However, with increasing DS the difference between *D*_h_ of UFH-PS-*cis* and UFH-PS-*trans* becomes more evident, with *D*_h_ of the chains substituted with PS-*cis* being smaller than those substituted with PS-*trans*. The size of Enox chains is clearly greater than that of UFH (about 50 nm) in spite of lower molecular weight. This may be due to the method of Enox production involving benzylation, which may result in some hydrophobization of Enox and the resulting tendency of its chains to aggregate. In contrast to UFH, substitution of Enox with PS resulted in an increase of *D*_h_ up to about 70 nm, with very little difference between chains with PS-*trans* and PS-*cis*, which may be ascribed to the much smaller size of the Enox chains. The conclusions drawn from DLS results were qualitatively confirmed with GPC measurements (see Figure S6 in the Supporting Information).

### Interaction with Azure A

**Figure 6:**
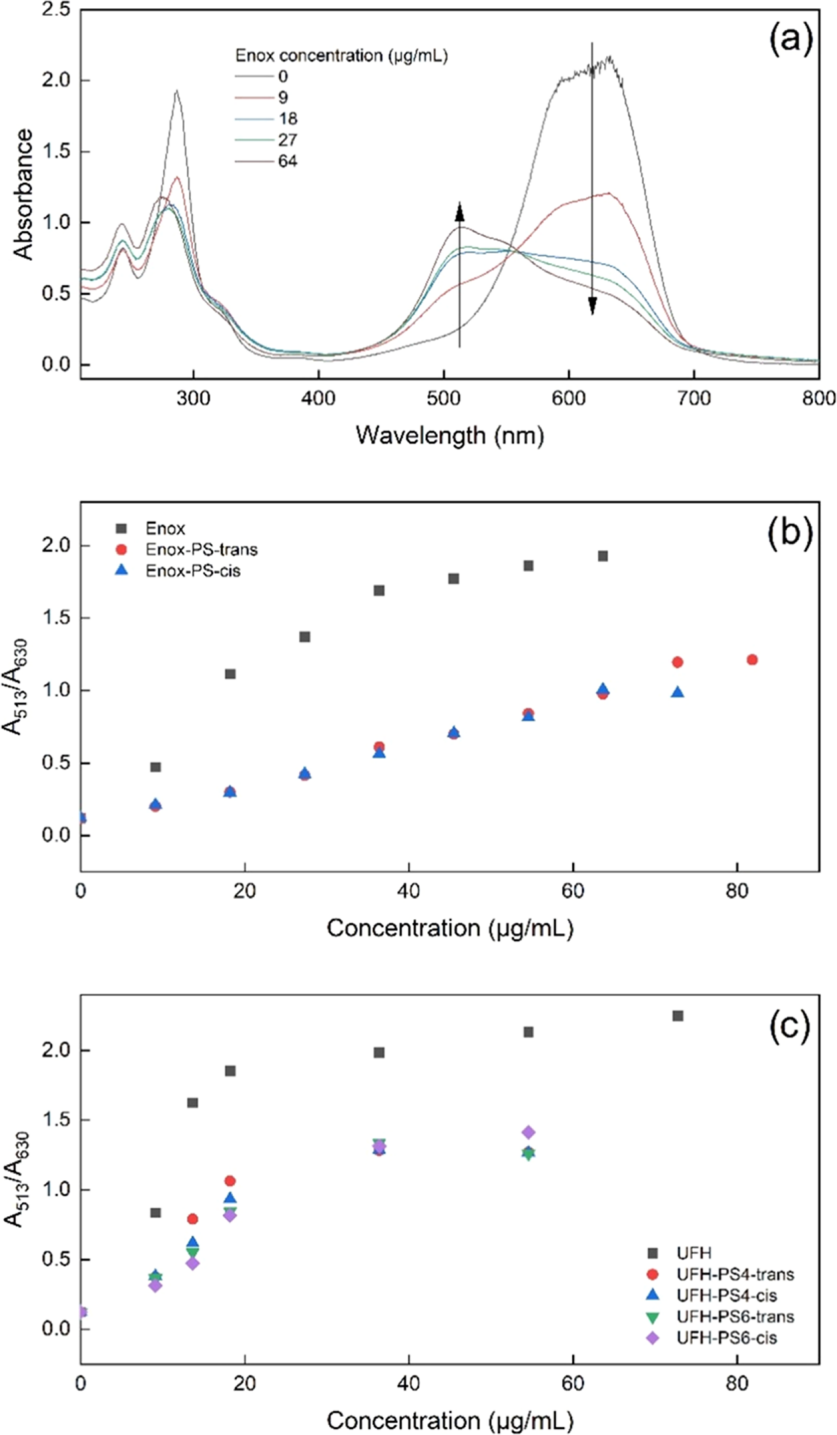
Changes in the spectra of Azure A with increasing Enox concentration (a) and the dependence of absorbance at 513 and 630 nm ratio (A_513_/A_630_) for Enox (b) and UFH and their versions modified with PS (c) in *trans* and *cis* configurations (*c*_Azure A_ = 6.67·10^–5^ mol/L).

It was verified if photoswitching changed the interaction of PS-substituted heparins with Azure A, a cationic dye, which is often used in quantitative heparin assays.^45^ In solution its molecules are attracted to negatively charged chains of heparin due to strong coulombic interactions and form aggregates along heparin chains. Aggregation is accompanied by an increase in the absorption band intensity at 513 nm and a decrease in the 630 nm band (see Figure 6a). Thus, the association of Azure A with heparin can be traced spectrophotometrically.^46,47^

The ability of Enox and UFH and their photoswitchable derivatives to complex Azure A was measured quantitatively as the ratio of the absorbance at 513 and 630 nm (A_513_/A_630_) (Figure 6b,c, respectively). For both substituted heparins, the ratio was smaller than that for unsubstituted ones, indicating that substitution with PS decreased the ability of heparins to complex Azure A. This may be because the substitution decreased the negative charge of the heparin chains by turning the negatively charged carboxyl groups into uncharged ester groups. Moreover, photoswitching of heparinic PS did not change binding of Azure A, neither by UFH-PS nor by Enox-PS.

### Anticoagulative Properties of Photoswitchable Heparins

The pentasaccharide sequence of heparin binds antithrombin (AT) primarily with sulfate groups attached to 3-*O* in unit III, to 6-*O* in unit I, and to 2-*N* in units III and V, while the carboxyl groups are not directly involved in AT binding.^48^ Thus, it could be expected that the anticoagulative properties of heparins esterified with PS are at least partially retained, as indicated by the literature.^40^ To verify this assumption and to find out if anticoagulative properties of UFH-PS and Enox-PS can be changed by photoswitching the attached PS, the aPTT was measured for murine plasma and lyophilized human plasma containing defined concentrations of UFH(-PS) and Enox(-PS), respectively (Table 3). The UFH derivative with the highest DS value (i.e., UFH-PS4) was selected for this compound, the greatest change of anticoagulative properties between *cis-* and *trans-*substituted UFH was expected based on DLS measurements.

**Table 3:** aPTT Times Measured at Various Concentrations of Both Photoisomers of Enox-PS and UFH-PS4 (*n* = 2)^a^

	aPTT ± SD (s)				
sample	0.005 mg/mL	0.025 mg/mL	0.05 mg/mL	0.075 mg/mL	0.10 mg/mL
control (PBS)	44 ± 1				
Enox		>180	>180	>180	>180
Enox-PS-*trans*		54 ± 4	67 ± 2	90 ± 9	124 ± 10
Enox-PS-*cis*		53 ± 3	65 ± 2	87 ± 3	120 ± 5
control (NaCl)	25 ± 1				
UFH	>180	>180	>180		
UFH-PS4-*trans*	46 ± 6	>180	>180		
UFH-PS4-*cis*	41 ± 8	>180	>180		

a aPTT for Enox and Enox-PS was found using lyophilized human plasma while for UFH and UFH-PS4 it was measured using murine plasma.

Enox-PS at a concentration as low as 0.025 mg/mL and UFH-PS4 at 0.005 mg/mL showed anticoagulative properties. This was indicated by the aPTT values of Enox-PS-*trans* and Enox-PS*-cis* equal to 54 ± 4 and 53 ± 3 s, respectively, compared to the shorter control value of 44 ± 1 s, while for UFH-PS4-*trans* and UFH-PS4-*cis*, the respective values were 46 ± 6 and 41 ± 8 s, compared to the shorter control value of 25 ± 1 s. At the same time aPTT for both modified heparins was significantly shorter than the corresponding values for Enox and UFH (both >180 s), indicating strong attenuation of anticoagulant activity of UFH-PS4 and Enox-PS compared to the parent heparins. The data for the range of concentrations studied do not show change in the anticoagulative activity of Enox-PS and UFH-PS4 upon photoswitching. For UFH-PS4 at concentrations ≥0.025 mg/mL the aPTT values for both photoisomers exceeded the measurement range of the coagulometer, so no change in aPTT upon photoswitching of UFH-PS4 could be found if any. Comparison of aPTT and DLS data for UFH suggests that in spite of the difference between the sizes of chains substituted with *trans* and *cis* photoisomers of PS, there is no difference in the anticoagulative properties between UFH-PS4-*trans* and UFH-PS4-*cis*.

### Interaction of Modified Heparins with Proteins

The interaction of UFH-PS6 with three proteins, i.e., human serum albumin (HSA), protamine, and lysozyme (Lys), in PBS at pH = 7.4 was assessed using ITC and compared with that of non-modified UFH. The model of a single set of binding sites was applied in all cases. The interactions between these three proteins and heparins were completely different. As expected based on the literature data for bovine serum albumin (BSA),^49^ the interaction between UFH and HSA was found to be too weak to be measured. There was no interaction between UFH-PS6-*trans* or UFH-PS6-*cis* and HSA, either. In the case of Lys, it was found to not interact with UFH while it showed a weak exothermal interaction with UFH-PS6 (Table 4). The entropy change was very low so the interaction was enthalpically driven. However, there were no discernible differences between the interaction of UFH-PS6-*trans* and UFH-PS6-*cis* with Lys.

**Table 4:** Thermodynamic Parameters of UFH and UFH-PS6 with Lysozyme

parameter	UFH	UFH-PS6-*trans*	UFH-PS6-*cis*
*n*	signal too weak to be measured	2.8 ± 0.1	2.4 ± 0.3
*K*_a_ (×10^6^ M^–1^)		0.7 ± 0.1	0.8 ± 0.1
Δ*H* (kJ/mol)		–35 ± 1	–35 ± 1
Δ*S* (J/mol/K)		–7 ± 3	–5 ± 4
Δ*G* (kJ/mol)		–33.3 ± 0.2	–33.9 ± 0.3
*K*_d_ (μM)		1.48 ± 0.14	1.18 ± 0.15

**Figure 7:**
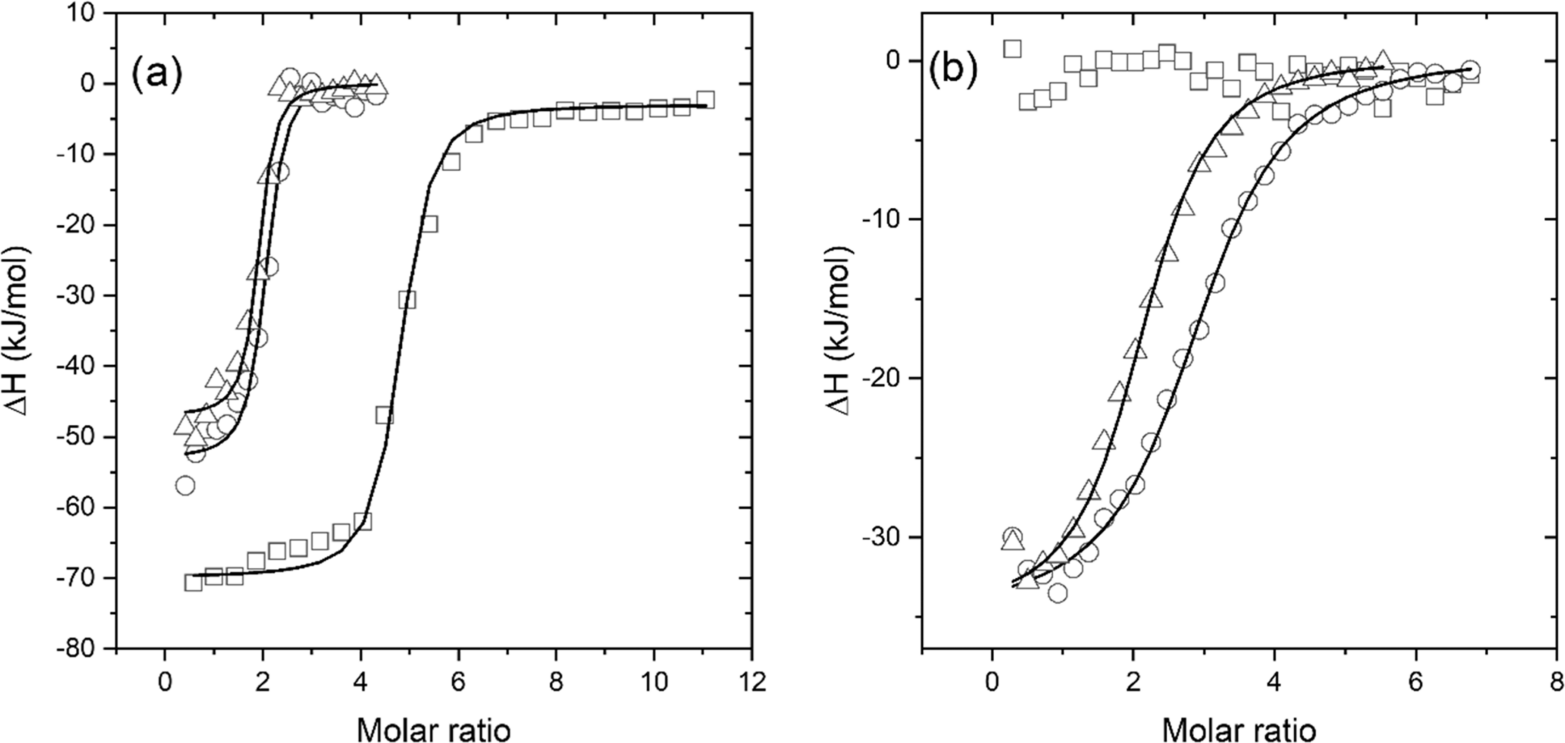
Representative ITC binding isotherms of heparin’s interactions with protamine (a) and lysozyme (b). The experiments were carried out in PBS pH 7.4 at 25 °C. UFH – squares, UFH-PS6-*trans* – circles, UFH-PS6-*cis* – triangles. Solid lines represent the best fit of one set of binding sites model to the data.

As expected for the oppositely charged polyelectrolytes, the heparins and protamine interacted strongly (Table 5). In this case, a noticeable difference was found between some thermodynamic parameters for the interaction of protamine with UFH and modified heparins. The number of binding sites for UFH was about 4 while for modified heparins it was close to 2, which suggests the important role of the UFH carboxylic groups in protamine binding (in UFH-PS these groups are substituted with a photoswitch, see Figure 3). On the other hand, the decrease of entropy for UFH-protamine interaction was much greater than that for interaction with modified heparin. In this case, there was also a significant difference in the entropy decrease between UFH-PS6-*trans* and UFH-PS6-*cis*, which for UFH-PS6-*trans* was twice as high as that for UFH-PS6-*cis*. The other thermodynamic parameters were similar for these three systems. The representative ITC binding isotherms of heparin interactions with protamine and lysozyme are shown in Figure 7

**Table 5:** Thermodynamic Parameters of UFH and UFH-PS6 with Protamine

parameter	UFH	UFH-PS6-*trans*	UFH-PS6-*cis*
*n*	4.4 ± 0.3	2.2 ± 0.2	1.7 ± 0.1
*K*_a_ (×10^6^ M^–1^)	10 ± 2	9 ± 3	13 ± 3
Δ*H* (kJ/mol)	–67 ± 1	–54 ± 2	–47 ± 1
Δ*S* (J/mol/K)	–91 ± 4	–48 ± 8	–22 ± 5
Δ*G* (kJ/mol)	–40 ± 0.4	–39.8 ± 0.8	–40.5 ± 0.6
*K*_d_ (μM)	0.1 ± 0.02	0.11 ± 0.03	0.08 ± 0.02

### Cytotoxicity

**Figure 8:**
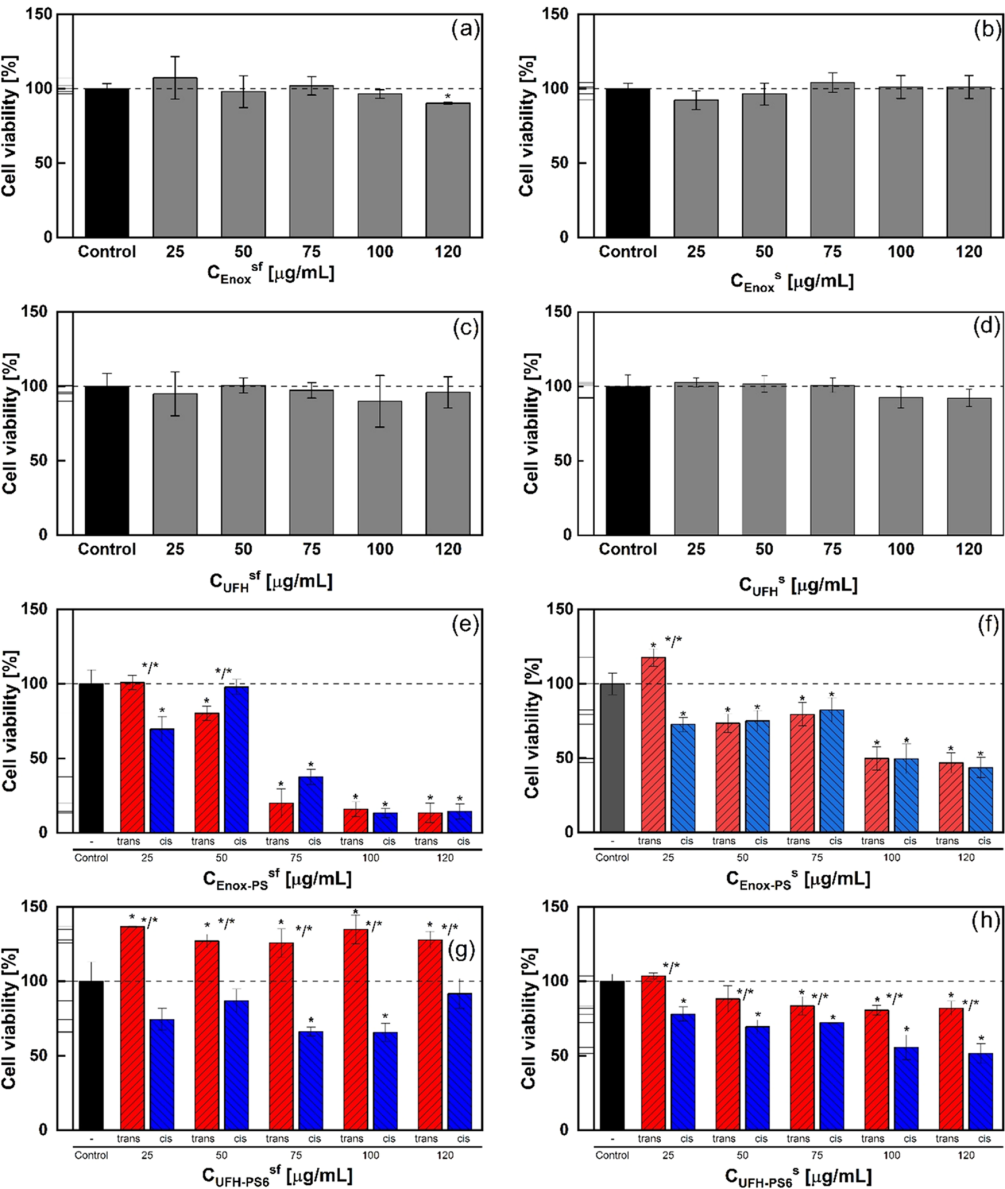
Influence on 3T3 cell viability of non-modified (Enox and UFH) and photoswitchable heparins (Enox-PS and UFH-PS6) in the absence (sf) and in the presence (s) of serum (Mann–Whitney test, *n* = 6, *p* = 0.05, error bars represent ±SD, * statistical difference from control, */* statistical difference between *trans* and *cis*). (a) and (b) Enox under serum-free and serum conditions, respectively; (c) and (d) UFH under serum-free and serum conditions, respectively; (e) and (f) Enox-PS under serum-free and serum conditions, respectively; and (g) and (h) UFH-PS under serum-free and serum conditions, respectively.

The cytotoxicity of photoswitchable heparins was tested on 3T3 mouse embryonic fibroblasts both under serum (s) and serum-free (sf) conditions (Figure 8). To increase the chance of observing a different behavior upon photoswitching, UFH-PS6 with a high degree of substitution with PS was tested. Neither unsubstituted Enox (Figure 8a,b) nor UFH (Figure 8c,d) were toxic for the selected cells under both conditions up to the concentration of 120 μg/mL. Only for Enox at 120 μg/mL a slight (by about 10%) statistically significant decrease of cell viability was found under serum-free conditions. However, the influence of both photosensitive heparins on 3T3 cell viability was quite different. Enox-PS decreased cell viability under both serum and serum-free conditions (Figure 8e,f, respectively), although under serum-free conditions this effect was more pronounced at higher concentrations. There were generally no statistically significant differences in cell viability for *trans* and *cis* forms of Enox-PS under both conditions. On the other hand, UFH-PS6-*trans* and UFH-PS6-*cis* showed different influences on cell viability both in the absence and in the presence of serum (Figure 8g,h, respectively). Under serum-free conditions, UFH-PS6-*trans* showed clear pro-proliferative activity, while UFH-PS6-*cis* moderately decreased cell viability, so both forms of UFH-PS6 had an opposite influence on 3T3 cell growth. On the other hand, in the presence of serum both forms of UFH-PS6 decreased the cell viability, although this effect for UFH-PS6-*cis* was statistically significantly higher than that for UFH-PS-*trans*. As opposed to the anticoagulative properties, the influence on cell viability of photosensitive heparins is correlated to the change of the chain size due to photoswitching, i.e., photoswitching of highly substituted UFH-PS results in changing both the chain size and cell viability, while no such differences were observed for Enox-PS.

## Experimental Section

### Reagents and Materials

Unfractionated heparin (UFH, Heparinum WZF, 5000 IU/mL, *M*_W_ 15 kDa, range of molecular weights 3–30 kDa. Polfa Warszawa S.A., Poland), low-molecular-weight heparin (LMWH, enoxaparin sodium, *M*_w_ 4.5 kDa, range of molecular weights 3.8–5.0 kDa, Suzhou Erye Pharmaceutical Co., Ltd.), lysozyme (BioShop), protamine (Sigma-Aldrich), human serum albumin (HSA, Sigma-Aldrich), 4-amino-1-methylpyrazole (Angene Chemical), phenol (Merck), dicyclohexylcarbodiimide (DCC, Sigma-Aldrich), benzethonium chloride (Hyamine 1622, Sigma-Aldrich), sodium acetate trihydrate (Chempur), sodium nitrite (Sigma-Aldrich), hydrochloric acid 35–38% (Sigma-Aldrich), sodium carbonate (Sigma-Aldrich), potassium carbonate (Sigma-Aldrich), potassium iodide (Sigma-Aldrich), sodium sulfate (Sigma-Aldrich), ethanol (Chempur), methanol (Fisher Scientific), dichloromethane (Chempur), acetonitrile (Fisher Scientific), DMF (Fisher Scientific), sulfuric acid (Sigma-Aldrich), Azure A, PBS (Sigma-Aldrich), dialysis tubes (MWCO 1 kDa, Carl Roth), and Bio-Rex 70 weakly acidic cation exchange resin (Bio-Rad). Deionized water was used in all experiments. All compounds are >95% pure by high-performance liquid chromatography (HPLC) analysis.

### Apparatus

Varian Cary 50 UV–VIS spectrophotometer (Agilent Technologies, Santa Clara, CA) was applied to record the spectra (transmittance mode, range: 200–800 nm, data interval: 0.5 nm), FT-IR spectrophotometer Nicolet iS10 (Thermo Scientific, Waltham, MA), and Nano ZS instrument (Malvern Instrument, Worcestershire, UK) were used. GPC measurements were performed using a Malvern Panalytical OMNISEC chromatograph. The PolySep-SEC GFC-P Linear column, LC Column 300 × 7.8 mm (Phenomenex, Torrance, CA) was used. The flow rate, injection volume, and polymer concentration were 0.8 mL/min, 100 μL, and 5 mg/mL, respectively, eluent: 0.1 M NaNO_3_ 80/20 H_2_O/acetonitrile. Irradiation of photoswitchable heparins was carried out using 340 nm (*trans–cis* isomerization) and 530 nm (*cis–trans* isomerization) LED lamps (Thorlabs). Maximum irradiance, *E*_e_, of the lamps was 0.6 and 9.46 μW/mm^2^, respectively, at a distance of 200 mm, as given by the manufacturer.

### ITC Measurements

ITC measurements were performed using a VP-ITC instrument (MicroCal, Northampton, MA) in PBS at pH 7.4. Measurements were taken at 25 °C, with a stirring speed of 300 rpm and an interval of 210 s between additions. The heparin solutions were placed in the cell and titrated with the protein solutions in 30 injections of 8–10 μL. The concentrations of the reagents in the UFH (or UFH-PS6)–protamine or HSA system were 5 and 300 μM, respectively, and in the UFH (or UFH-PS6)–lysozyme system they were 10 and 300 μM, respectively. The molar concentration of UFH (and UFH-PS6) and protamine was calculated assuming their molecular weights of 15 and 4.5 kDa, respectively. The molar concentration of human serum albumin (HSA) and lysozyme (Lys) was calculated based on their molar absorption coefficients of 35 700, and 37 860 M/cm, respectively. Analyses were performed globally for at least two measurements according to a one-set binding site model with shared values of *K*_a_ and ΔH.

### Irradiation of PS and Heparin Solutions

The PS, UFH-PS, and Enox-PS solutions in aqueous media were irradiated in 1-cm quartz cuvettes under constant mixing. The intensity of the light at the distance from the lamps, at which the cuvettes were held (about 5 cm), was 0.328 mW/cm^2^ for a 340 nm LED and 4.96 mW/cm^2^ for a 530 nm LED.

### UPLC-MS Analysis

The UPLC-MS/MS system consisted of a Waters ACQUITY UPLC (Waters Corporation, Milford, MA) coupled to a Waters TQD mass spectrometer (electrospray ionization mode ESI-tandem quadrupole). Chromatographic separations were carried out using the Acquity UPLC BEH (bridged ethylene hybrid) C18 column; 2.1 × 100 mm, and 1.7 μm particle size, equipped with an Acquity UPLC BEH C18 VanGuard pre-column; 2.1 × 5 mm, and 1.7 μm particle size. The column was maintained at 40 °C, and eluted under gradient conditions using 95 to 0% of eluent A over 5 min, afterward isocratic elution using 100% of eluent B over 5 min, at a flow rate of 0.3 mL/min. Eluent A: water/formic acid (0.1%, v/v); eluent B: acetonitrile/formic acid (0.1%, v/v). Chromatograms were recorded using a Waters eλ PDA detector. Spectra were analyzed in the 200–700 nm range with 1.2 nm resolution and a sampling rate of 20 points/s. MS detection settings of Waters TQD mass spectrometer were as follows: source temperature of 150 °C, desolvation temperature of 350 °C, desolvation gas flow rate of 600 L/h, cone gas flow of 100 L/h, capillary potential of 3.00 kV, and cone potential of 30 V. Nitrogen was used as both nebulizing and drying gas. The data were obtained in a scan mode ranging from 50 to 1000 m/z at 0.5 s intervals. Data acquisition software was MassLynx V 4.1 (Waters).

### Synthesis of the Photoswitch (PS)

The photoswitch was synthesized using a modified literature procedure.^38^ 4-Amino-1-methylpyrazole (**1**, 2.91 g, 30 mmol, 1 equiv) was dissolved in 60 mL of water, followed by the addition of 14 mL of HCl (12.2 mol/L, 170 mmol). After the solution was cooled to 0–5 °C, a prechilled solution of NaNO_2_ (2.7 g, 39 mmol, 1.3 equiv) in 60 mL water was slowly added. After the mixture was stirred for 30 min in a 0 °C bath, a prechilled solution of phenol (3.38 g, 36 mmol, 1.2 equiv) and NaOH (3.24 g, 80 mmol) in 100 mL of water was slowly added. Then, a prechilled solution of Na_2_CO_3_ (10.6 g, 100 mmol) in 80 mL of water was slowly added and yellow-brown particles were formed. The reaction mixture was stirred for 1 h. The resulting suspension was filtered out and washed with water. The filter cake was dried to give **2** as a yellow solid (4.50 g, 74%). To a mixture containing **2** (1.00 g, 4.9 mmol, 1 equiv), K_2_CO_3_ (2.73 g, 19.8 mmol, 4 equiv), KI (41 mg, 0.2 mmol, 0.05 equiv) in 9 mL of DMF was added 1-bromo-2-ethanol dropwise (0.95 mL, 13.4 mmol, 2.7 equiv). The solution was then stirred at 110 °C under reflux for 3 h. The reaction mixture was quenched by adding water and stirred for 30 min to crystallize solid PS which was then filtered out and washed with water. The filter cake was dissolved in ethyl acetate, and the solution was dried over Na_2_SO_4_ and concentrated under reduced pressure. Purification by column chromatography (hexane/ethyl acetate 4:1) afforded PS as yellow crystals (920 mg, 75%). Purity: 97.8% (HPLC, see Figure S1). Elemental analysis (%): C: 58.59 (theor. 58.53), H: 5.75 (theor. 5.73), N: 22.34 (theor. 22.75).

### Synthesis of the Photoswitchable Heparins

#### Photoswitchable Unfractionated Heparin (UFH-PS)

The synthesis was performed in 3 steps involving (1) the synthesis of UFH ammonium salt according to the modified patent procedure,^39^ (2) exchange of Na^+^ to H^+^ ions,^39^ and (3) esterification of the UFH carboxyl groups with PS according to modified patent procedure^40^ (Figure S1 in the Supporting Information, SI). The example of the synthesis procedure was as follows. Commercial UFH was purified by dialysis against water and lyophilized. Then, 1 g of UFH was dissolved in 1 L of water. In this solution, 2.7 g of benzethonium chloride (Hyamine 1622) was dissolved and 13 mL of 0.5 M H_2_SO_4_ was added. The mixture was left overnight and centrifuged (10 000 rpm, 10 min). The precipitate was washed 3 times with 20 mL aliquots of water and the product was dried *in vacuo* for one week. Then, 2.126 g of the ammonium salt of UFH (**3**) was obtained; 2 g of **3** was dissolved in 70 mL of ethanol. To this solution 2.4 g of the acidic form of the Bio-Rex 70 resin was added, and the mixture was stirred for 30 min, then the resin was filtered out. After evaporation of ethanol under reduced pressure 2.332 g of ammonium salt **4** was obtained. To the solution of 250 mg of **4** in 1 mL of DMF, 20 mg of PS, and 594 mg of DCC in 5 mL of DMF were added. The mixture was left for 2 days at 4 °C. The solution was filtered. To precipitate the product, 5 mL of 95% ethanol and 5 mL of 10% w/v sodium acetate solution in MeOH were added to the filtrate. The product (UFH-PS-*trans*) was centrifuged (5000 rpm, 5 min) and then washed five times with 5 mL of EtOH. Next, the precipitate was dissolved in 15 mL of water and purified with dialysis against distilled water for 2 weeks. UFH-PS-*trans* was isolated from the solution by lyophilization (44 mg).

#### Photoswitchable Enoxaparin (Enox-PS)

The ammonium salt of Enox was obtained in a similar way as UFH-PS, using 2.0 g of enoxaparin (Enox) and proportional amounts of other reagents. Then, 4.82 g of the ammonium salt of Enox (**3**) was obtained and 4.72 g of **3** was dissolved in 155 mL of ethanol and mixed overnight. To this solution 5.33 g of the acidic form of Bio-Rex 70 resin was added, and the mixture was stirred for 1 h. Then the resin was filtered out and the solvent was evaporated and 4.11 g of **4** was obtained. Then, 2.0 g of PS and 2.38 g of DCC were dissolved in 10 mL of DMF and the solution of 1.02 g of **4** in 4 mL of DMF was added. The mixture was left in ice for 3 days at 4 °C and filtered. To precipitate the product 30 mL of 95% ethanol and 30 mL of 10% w/v sodium acetate solution in MeOH were added to the filtrate. The product (Enox-PS-*trans*) was centrifuged (5000 rpm, 5 min) and then washed with DCM until the filtrate was colorless.

### Cytotoxicity/Proliferation Tests

3T3 L1 murine fibroblasts (ATCC), fetal bovine serum (FBS, Sigma-Aldrich), crystal violet (CrV, Sigma-Aldrich), formaldehyde (Sigma-Aldrich), DMEM high-glucose (Sigma-Aldrich), and destaining solution (0.065 M citric acid, 0.04 sodium citrate in MeOH/H_2_O 1:1) were used. The influence of photoswitchable heparins on cell viability was tested on 3T3 mouse embryonic fibroblasts. 3T3 L1 cells were grown in Petri dishes in DMEM supplemented with 10% (v/v) FBS, at 37 °C in a humidified atmosphere containing 5% (v/v) CO_2_. Next, the cells were seeded in 48-well plates and grown for 24 h. After that, in the serum-free experiment, the medium with 10% FBS was changed to a medium without FBS. Cells were treated with 50 μL of photoswitchable heparins with PS both in *trans* and *cis* forms in PBS (*cis* form was obtained by irradiation of the *trans* forms with 340 nm light directly before the experiment) at 5 different concentrations and incubated for 24 h. To the control, 50 μL of PBS was added. To assess cell viability, the crystal violet (CrV) assay was used. After incubation, the medium was removed, and the cells were washed with 0.5 mL of PBS, next mixed using 0.5 mL of 4% v/v formaldehyde/PBS and left for 10 min, washed again with 0.5 mL of PBS and treated for 2 min with a CrV solution. Then, unbound CrV was removed by rinsing with water. After drying, the destaining solution was added to each well and left for 20 min. Finally, the absorbance of the obtained solution at 540 nm was measured, which was proportional to the number of living cells.

### Coagulation Tests

Murine or lyophilized human plasma, Dia-PTT and Dia-CaCl2 reagents (both Diagon Ltd.), were used in the tests. The heparins substituted with PS-*cis* were obtained by irradiation of the respective *trans* forms with 340 nm light directly before the experiment. To 100 μL of murine plasma 10 μL of the solution of the appropriate heparin or NaCl or PBS were added and incubated for 5 min at 37 °C. Next, 50 μL of the obtained sample was placed in a cuvette in the coagulometer at 37 °C and 50 μL of the Dia-PTT reagent was added. The sample was incubated for 3 min at 37 °C. Then, 50 μL of the Dia-CaCl2 reagent was added and aPTT was measured with a coagulometer (Coag 4D, Diagon Ltd.).

## Conclusions

The photosensitive derivatives of both UFH and LMWH were obtained by substitution with a PS able to undergo quantitative *trans–cis* and reverse photoisomerizations. The thermally unstable *cis* photoisomer of the PS attached to heparins showed an exceptionally long half-life in the aqueous media at 37 °C enabling investigation if photoswitching of the PS attached to heparin chains may result in the change in any physicochemical and biological properties of this biopolymer. It was found that substitution with PS attenuated the anticoagulant properties of both UFH and Enox. Both Enox-PS-*trans* and Enox-PS*-cis* showed similar cytotoxicity at higher concentrations, whereas they had no significant effect on the cell viability at concentrations prolonging aPTT. On the other hand, under serum-free conditions, UFH-PS-*trans* stimulated cell proliferation, while UFH-PS*-cis* decreased cell viability. Thus, the data obtained indicate that it is possible to gain photocontrol over some of the biological activities of heparin even if its degree of substitution with a PS is rather small, while simultaneously decreasing its anticoagulant activity, which may open new applications for this drug.

## Supporting Information Available

Figure S1. LC-MS data of PS (PDA detector); Figure S2. Scheme of UFH-PS and Enox-PS synthesis; Figure S3. FT-IR absorption spectra of Enox, Enox-PS, and PS; Figure S4. ^1^H NMR spectrum of 2; Figure S5. ^1^H NMR spectrum of PS; Figure S6. ^13^C NMR spectrum of PS; Figure S7. GPC traces of UFH-PS6 and Enox-PS; Figure S8. Spectral distributions of the LED lamps used for the irradiation (PDF)

## Abbreviations

AAP: arylazopyrazole
aPTT: activated partial thromboplastin time
DENV: dengue virus
DLS: dynamic light scattering
*D*_h_: hydrodynamic diameter
DMEM: Dulbecco’s Modified Eagle’s Medium
DS: degree of substitution
HIV: human immunodeficiency virus
HPV: human papilloma virus
HSV-1: herpes simplex virus 1
IV: influenza virus
LMWH: low-molecular-weight heparin
MWCO: molecular weight cut-off
PS: photoswitch
UFH: unfractionated heparin
ZIKV: Zika virus
