# Supplementary material for: Biological Properties of Heparins Modified with an Arylazopyrazole-Based Photoswitch
Source: J Med Chem. 2023 Jan 19;66(3):1778–89. doi: 10.1021/acs.jmedchem.2c01616 (PMC9923745; doi:10.1021/acs.jmedchem.2c01616)
Supplement: Supplementary file 1 — jm2c01616_si_001.pdf [file jm2c01616_si_001.pdf]

Supporting Information

Biological properties of heparins modified with an arylazopyrazole-based photoswitch

Marta Stolarek[1], Aleksandra Pycior[1], Piotr Bonarek[2], Małgorzata Opydo[3], Elzbieta Kolaczkowska[3], Kamil Kamiński[1], Andrzej Mogielnicki[4], Krzysztof Szczubiałka[1,*]

[1] *Faculty of Chemistry, Jagiellonian University, Gronostajowa 2, 30-387 Krakow, Poland*

[2] *Faculty of Biochemistry, Biophysics and Biotechnology, Jagiellonian University, Gronostajowa 7, 30-387 Krakow, Poland*

[3] *Laboratory of Experimental Hematology, Institute of Zoology and Biomedical Research, Faculty of Biology, Jagiellonian University, Gronostajowa 9, 30-387 Krakow, Poland*

[4] *Department of Pharmacodynamics, Medical University of Bialystok, Mickiewicza 2c, 15-089 Bialystok, Poland*

Corresponding author email: szczubia@chemia.uj.edu.pl

Contents

Figure S1. LC-MS data of PS (PDA detector)

Figure S2. Scheme of UFH-PS and Enox-PS synthesis

Figure S3. FT-IR absorption spectra of Enox, Enox-PS and PS

Figure S4. ^{1}H NMR spectrum of 2.

Figure S5. ^{1}H NMR spectrum of PS

Figure S6. ^{13}C NMR spectrum of PS

Figure S7. GPC traces of UFH-PS6 and Enox-PS.

Figure S8. Spectral distributions of the LED lamps used for the irradiations.

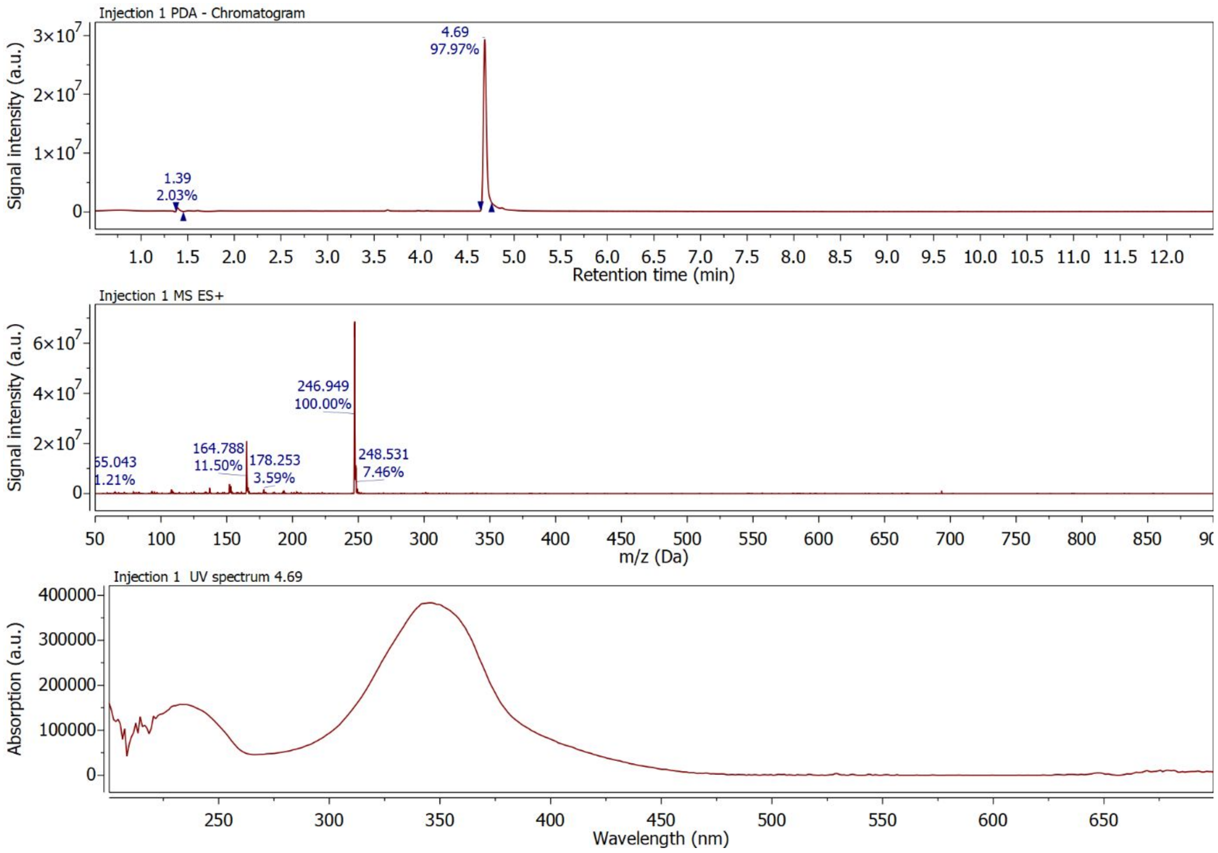

Figure S1. LC-MS data of PS (PDA detector)

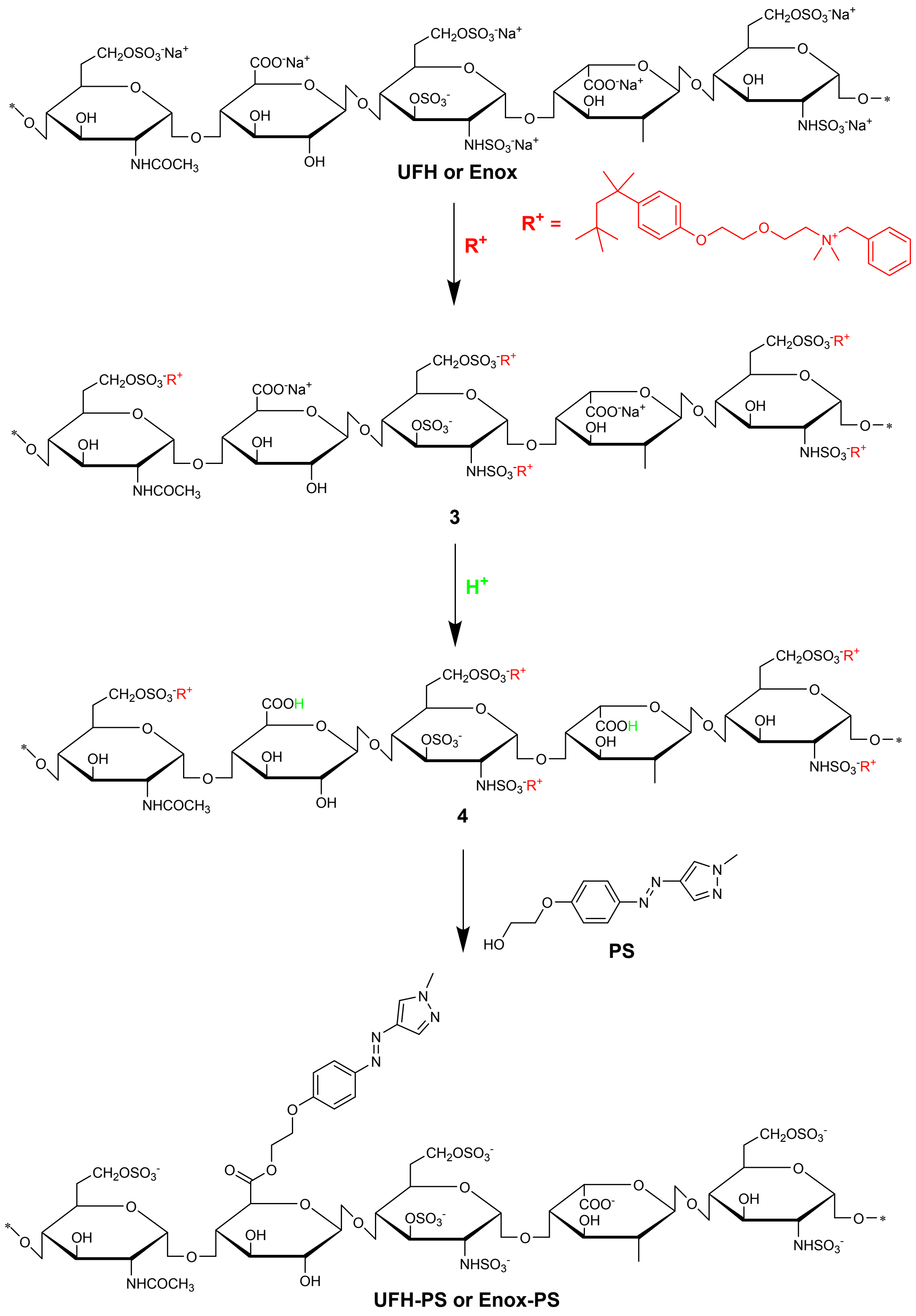

Figure S2. Scheme of UFH-PS and Enox-PS synthesis

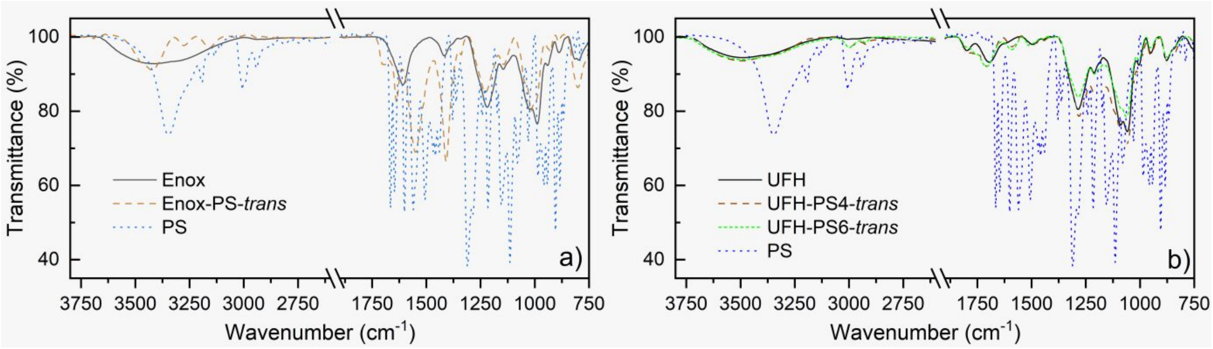

Figure S3. FT-IR absorption spectra of (a) Enox, Enox-PS-*trans* and PS; and (b) UFH, UFH-PS4-*trans*, UFH-PS6-*trans*, and PS

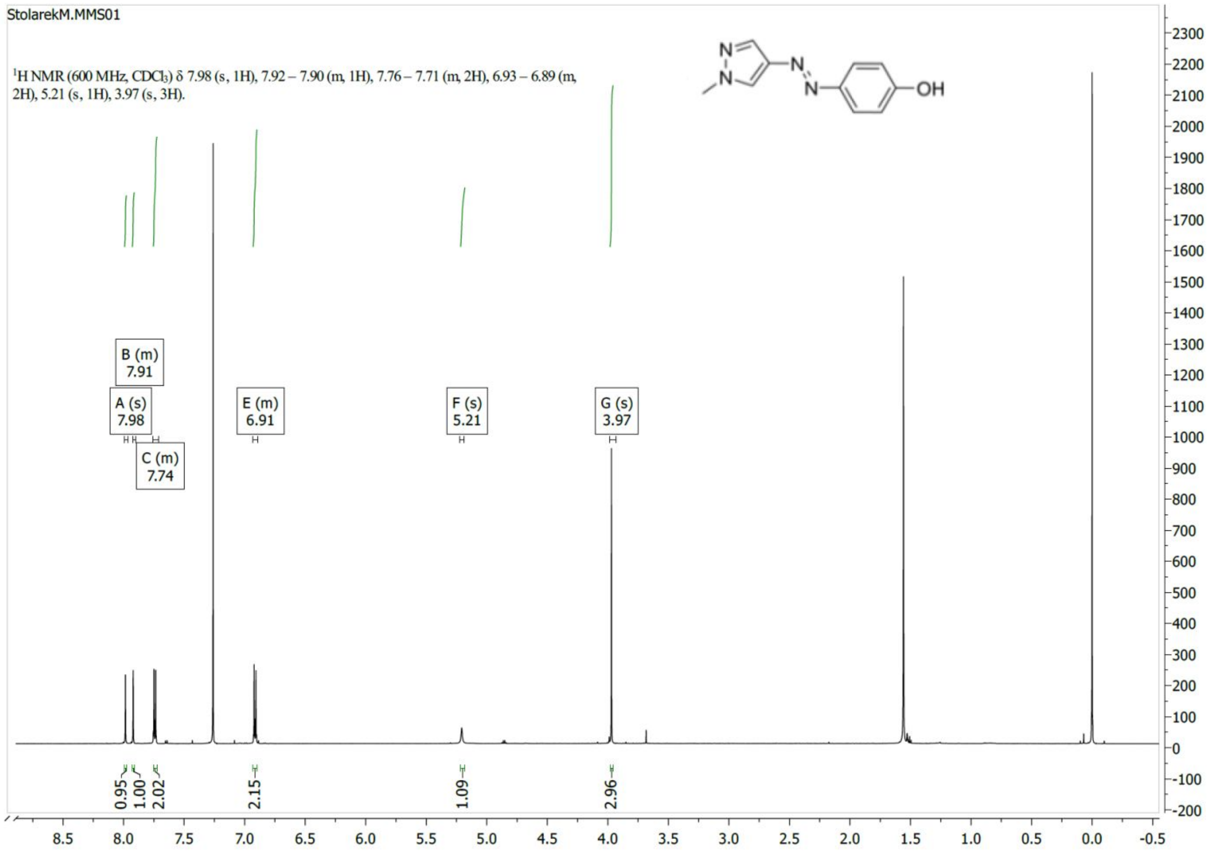

Figure S4. ^{1}H NMR spectrum of 2.

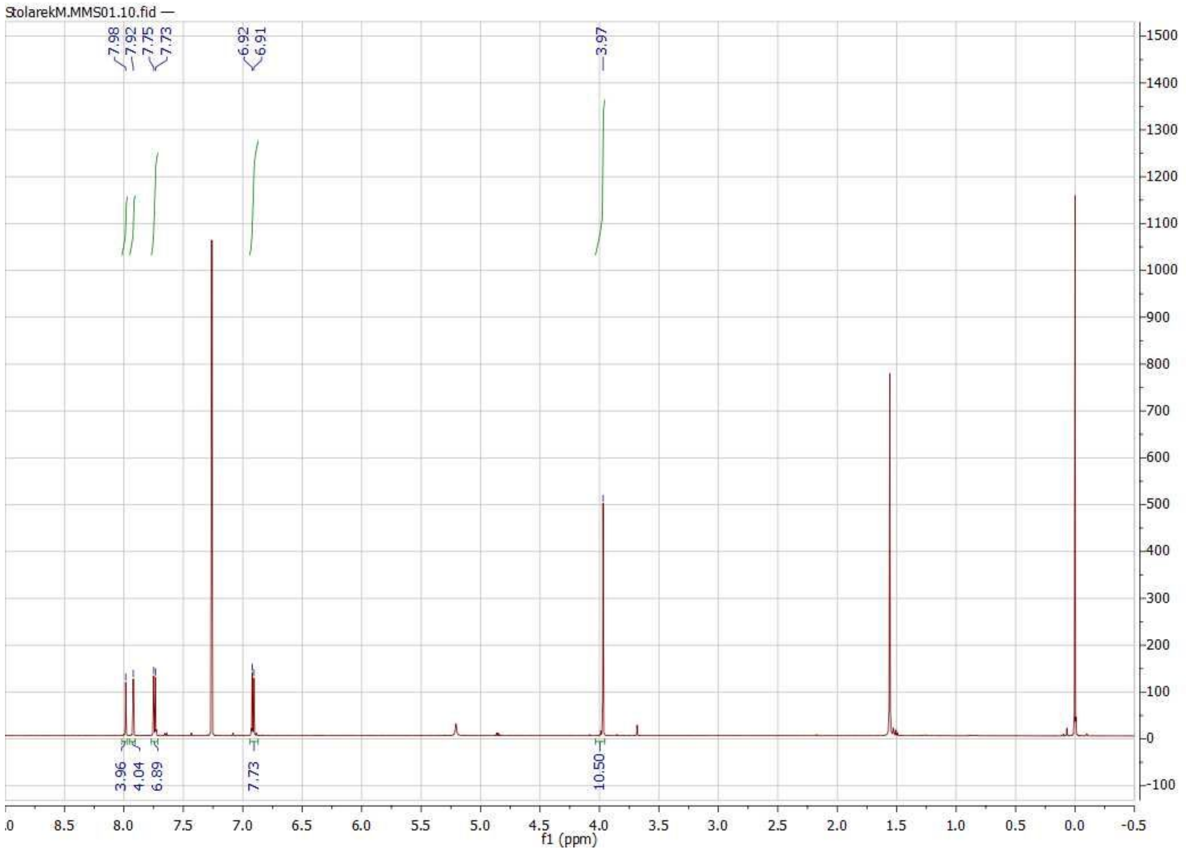

Figure S5. **^{1}H NMR spectrum of PS**

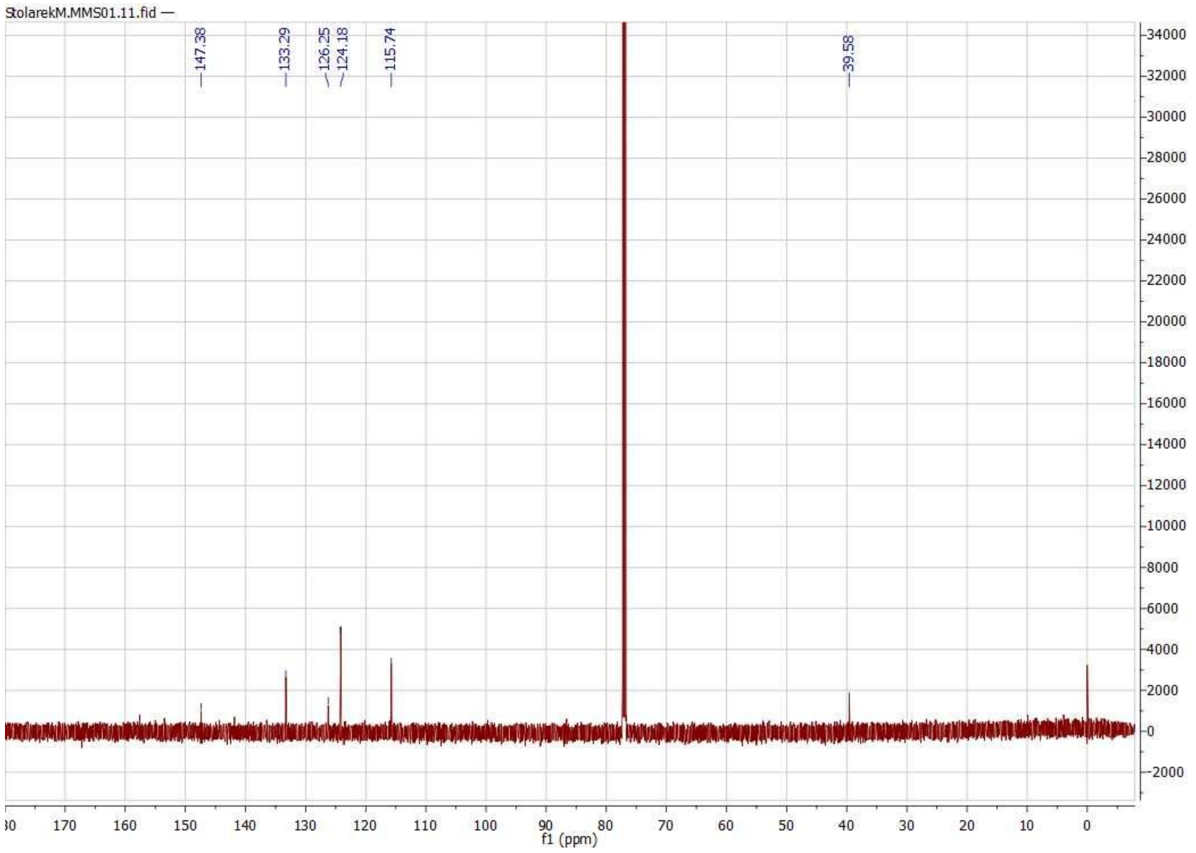

Figure S6. ^{13}C NMR spectrum of PS

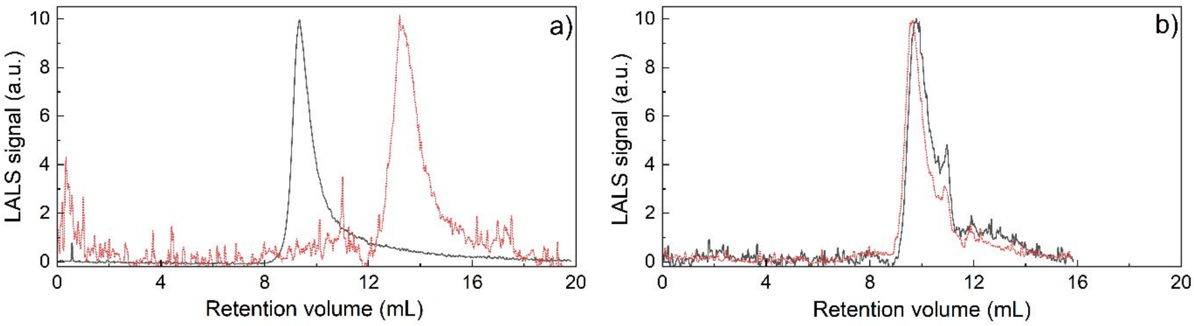

Figure S7. GPC traces of a) UFH-PS6 and b) Enox-PS. Traces for *trans* and *cis* PS configuration are shown a solid and dotted lines, respectively.

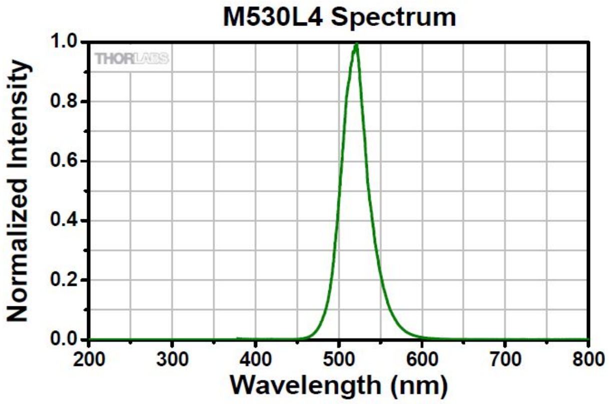

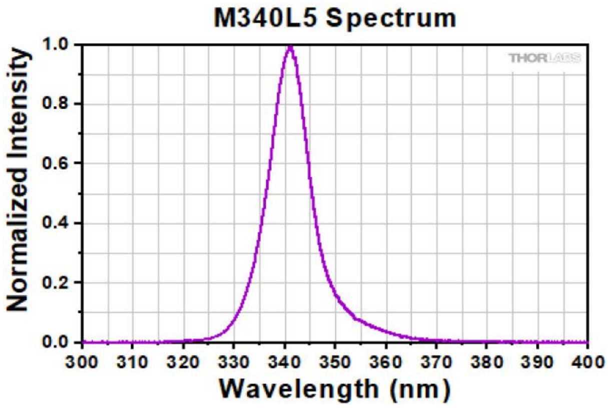

Figure S8. Spectral distributions of the LED lamps used for the irradiations. Data taken from the lamp specifications given by the maufacturer (Thorlabs, www.thorlabs.com).
